# Finding the missing many: improving Tuberculosis care in Kajiado county through active case finding and community approaches

**DOI:** 10.1186/s12889-023-17631-2

**Published:** 2024-01-10

**Authors:** Gianfranco Morino, Caleb Mike Mulongo, Paolo Cattaneo, Maria Vittoria De Vita, Gabriele Paone, Simone Scarlata, Federico Gobbi, Salome Kinyita, Hillary Odhiambo

**Affiliations:** 1 World Friends Onlus, Nairobi, Kenya; 2 Health Systems Unit, Tunu Consulting Hub, Nairobi, Kenya; 3 Department of Infectious, Tropical Diseases and Microbiology, IRCCS Sacro Cuore Don Calabria Hospital, Negrar di Valpolicella, Verona, Italy; 4 Unit of internal medicine, Respiratory pathophysiology and thoracic endoscopy, Fondazione Policlinico Universitario Campus Bio Medico, Rome, Italy

**Keywords:** GeneXpert, Tuberculosis, Active case finding, Patient-centred Tuberculosis care, Decentralized Tuberculosis care

## Abstract

**Background:**

Tuberculosis related deaths remain a priority globally. Despite advancements in TB care, access to quality care remains inequitable to the disadvantage of those in rural and urban informal settlements. The Awareness, Traditions, and Innovation in combating Tuberculosis (ATI TB) project incorporated active case finding (ACF), use of GeneXpert technology and decentralized services to improve TB care in Kajiado County. This study sought to establish the impact of the project as well as implementation lessons learnt during its tenure in Kajiado County, Kenya.

**Methods:**

This evaluation adopted a mixed-methods approach with retrospective cohort analysis for the quantitative data and qualitative data sought through key informant interviews with 28 purposively sampled respondents. The qualitative data was analyzed thematically using *Taguette* while quantitative data was analyzed using *R* Software yielding descriptive statistics and measures of association.

**Results:**

While the males were a minority among the presumptive cases (623; 46%), they were the majority (59.3%) among the confirmed TB cases. 70% of the confirmed cases were aged between 15 and 44 years; with those aged between 25- and 34-years being majority (30% of the cases). Majority of the confirmed cases within the project were from rural Kajiado West (79; 66.9%). Though 61% of the presumptive cases were through ACF, only 7% of these tested positive. Conversely, 13% of the self-referrals tested positive. 53% (66) of the positive cases with valid data were self-referrals while ACF accounted for 47% (58) of the positives.

**Conclusion:**

Continued capacity development among health workers, sustained and targeted sensitization and screening among vulnerable groups, strategic collaborations, alongside increased budgetary prioritization of health and TB care by government and partners, and government investments in Social Determinants of Health can ensure gains in TB care are sustained.

**Supplementary Information:**

The online version contains supplementary material available at 10.1186/s12889-023-17631-2.

## Key messages

a. The higher and rising incidence of TB (All forms) among the males compared to females in Kajiado highlights the need for sustained sensitization, active case finding and care for all genders but more targeted to the males in Kajiado County.

b. Active Case Finding increases the potential for identification of Drug Resistant TB

c. The rising cases of relapses among patients with Extra-Pulmonary Tuberculosis in Kajiado County necessitates increased attention, awareness creation, heightened sensitization on Extra-Pulmonary TB, capacity development among health workers for improved diagnosis, care, and treatment for extra-pulmonary TB alongside improved adherence for respective patients.

d. The higher number of positive TB cases among the self-referrals (walk- in patients) compared to those identified via active case finding underscore the reality of unmet care needs at community level, most of which are underdiagnosed and under reported.

e. Integrated and Patient centered care approaches that not only target TB but address other determinants of health (socio-cultural, economic, environmental, political) are more likely to yield better engagement, ownership, impact and sustainability.

**Supplementary Information:**

The online version contains supplementary material available at 10.1186/s12889-023-17631-2.

## Introduction

### Background

Tuberculosis related deaths remain a priority globally. It is estimated that about a quarter of the world’s population is infected with *Mycobacterium tuberculosis* hence at high risk of developing TB disease [1]. It is a curable disease, and its disease burden can be reduced with improved access to diagnosis and treatment.

In Kenya, there was more than twice as much TB as previously estimated at 426 cases per 100,000 population [2–4]. About two thirds of the patients with TB symptoms in Kenya are yet to seek treatment. 64% of TB patients had been detected, notified, and commenced on first-line treatment whilst 36% were missed by the surveillance system. The notified cases of Drug Resistant TB increased by 50% between 2017 and 2018 [5].

The mortality rate of TB in Kenya (60/100,000) is triple the global average [6] in spite an estimated 83% TB treatment success rate [2, 7]. About 0.02% of the population in Kajiado County were diagnosed to have TB in 2019/20 with an estimated HIV/TB co-infection rate of 29% [8].

Symptomatic screening of TB has previously been based on four cardinal symptoms: cough of more than two weeks, fever, night sweats and weight loss. Unfortunately, this has been associated with the missing of more than 40% of TB cases [9]. This necessitated the expansion of the criteria to include any TB related symptoms - cough of any duration, night sweats, weight loss, fatigue, fever, and shortness of breath [6, 9]. Coupled with broader use of chest radiographs for all presumed TB cases, this further increases the screening yield for TB cases.

Conventional microscopy misses about 50% of TB cases but the use of GeneXpert, a cartridge based nucleic acid amplification test, increases the efficiency of diagnosis as it detects 78% of TB cases [10]. It is thus the recommended first line diagnostic test for all presumed TB cases [6, 9]. There is limited use of the technology across the country, particularly in the remote and rural settings. Notably, Kajiado County only had 3 GeneXpert machines prior to commencement of this project.

In most countries with a high TB burden, private health providers remain a critical link to care and treatment yet are often not linked to the public health system [11–13]. Private healthcare utilization in Kenya is estimated at 41%, most of it being in lower tier facilities [14, 15]. About a third of the TB patients seek care in private facilities [9] and only 43% of all patients had access to a facility with adequate diagnostic capacity or specimen transport [2]. Only 42% of health facilities in Kenya offer TB care. Of these, only 31% offered any form of diagnosis; and 25% offered diagnosis by sputum microscopy assessment [16].

Only 24% of the facilities provide any services to drug resistant TB patients. TB services are more available in public primary facilities (98%) and least available in private facilities (15%) [16]. Private facilities account for 48% of health facilities in the country [15, 17]. Despite the presence of private facilities, majority lack clear systems of linking patients to appropriate TB diagnosis and care [14] whilst the country has a fairly robust referral system for linkage of remote public facilities to centralized TB care facilities [2]. This shows the potential the private sector has in bridging the diagnostic and care gap in the fight against TB [9, 18–20].

Patient-centered approaches to TB care are crucial in improving adherence and outcomes, recognizing and respecting the individual and community health needs, values, preferences, and expectations beyond the medical requirements of the disease [20–25] The World Health Organization's End TB Strategy recommends social and tailored treatment and adherence support, including community- or home-based treatment provided by trained health workers or lay providers [25, 26]. Decentralized care is also recommended as it allows for easier access to treatment and counseling, potentially reducing the financial burden of TB disease and enabling patients to remain with their families [23, 27, 28]

The Awareness, Tradition, and Innovation in combating Tuberculosis “ATI-TB” project leveraged a) existing practices, information, and attitudes on TB; b) use of GeneXpert Technology; and c) decentralized care to improve TB care in Kajiado County. The project sought to increase:

1. Awareness and demand for TB care among the rural and urban populations through outreaches and deployment of Community Health Volunteers (CHVs)
2. Screening, diagnosis using GeneXpert for bacteriological confirmation, and treatment of TB in the urban key populations, remote and rural areas
3. Knowledge of the socio-cultural context of Kajiado County and how it influences TB care.

The project partnered with selected public and private facilities in Kajiado county, donated two GeneXpert machines to one public and one private facility; deployed CHVs and TB educators to the communities, held screening and care outreaches and facilitated sample referral within the county.

This end term study sought to establish and document the effect of the project on TB diagnosis and care as well as implementation lessons learnt during the tenure of the project in Kajiado County between May 2021 and January 2023.

### Conceptual framework

**Fig. 1:**
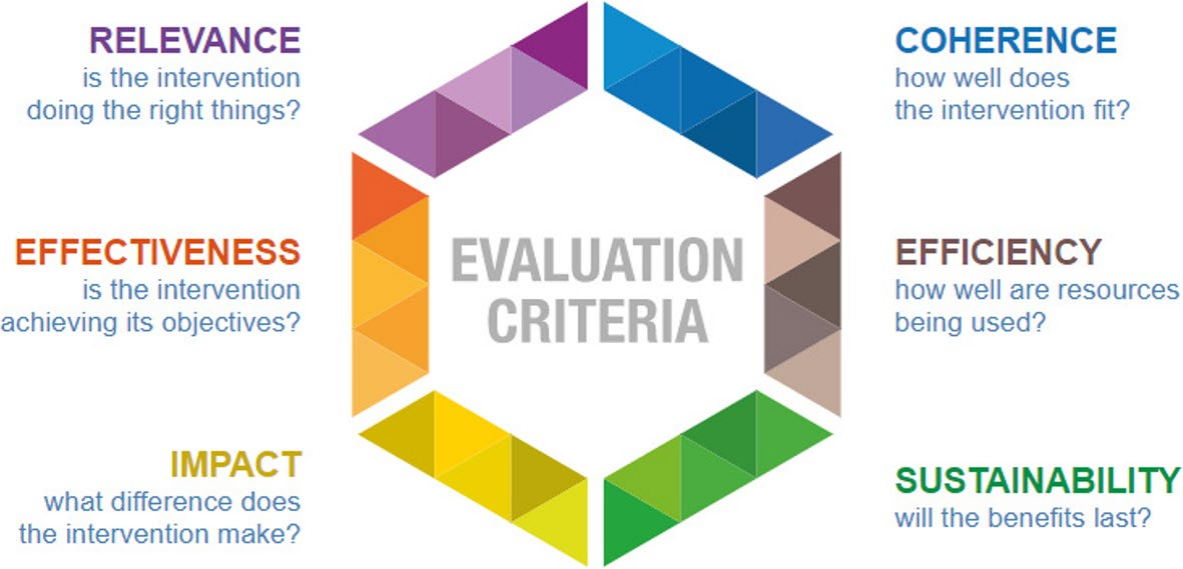
OECD DAC Network on Development Evaluation (EvalNet) Framework

Utility of the project during its tenure was evaluated using the Organisation for Economic Co-operation and Development's Development Assistance Committee (OECD DAC) Network on Development Evaluation (EvalNet) framework [29] in Fig. 1. This allowed for a broad assessment of the project processes and outcomes in line with six dimensions; relevance, coherence, effectiveness, efficiency, impact and sustainability. Additional focus was centred on equity and participation.

## Methods

This end-term study was a retrospective observational study among beneficiaries, and stakeholders of the ATI TB project within Kajiado County. This evaluation adopted a mixed methods approach to draw insights from the respective stakeholders and data repositories.

### Study site

The study was conducted in four sub-counties in Kajiado County, where the project was implemented, including Kajiado North, Kajiado East, Kajiado Central and Kajiado West.

### Population, sampling and recruitment

The study population consisted of all project beneficiaries, key community members, and county healthcare stakeholders in Kajiado County. The study adopted non-random sampling techniques in the selection of respondents. Consequently, purposive sampling and snow-ball sampling were used to select twenty-eight [28] respondents from among the beneficiaries of the project and key stakeholders with information about the communities, TB care and the project (Table 1).

**Table 1:** Summary of respondents and data collection methods

**Respondent**	**Sampling**	**Data Collection Strategy**	**Number**
Government Representatives	Purposive - Familiarity with TB Policies and Care	Key Informant Interviews	4
Screened Patients	Purposive & Snowball- Project Beneficiaries	Key Informant Interviews	5
Patients on Treatment	Purposive & Snowball- Project Beneficiaries	Key Informant Interviews	8
Urban Community Representatives	Purposive- Familiarity with the project and TB care in the community	Key Informant Interviews	2
Rural Community Representatives	Purposive- Familiarity with the project and TB care in the community	Key Informant Interviews	2
Partner Representatives	Purposive- Familiarity with the project and TB policies and care in Kajiado	Key Informant Interviews	2
Project Representatives	Purposive- Familiarity with the project goals and implementation.	Key Informant Interviews	2
Health Workers	Purposive- Familiarity with the project and TB policies and care in Kajiado	Key Informant Interviews	3

### Clinical evaluation of subjects

Presumptive cases were identified through screening using the protocol recommended by Kenyan Integrated Guidelines for TB (2021) in the outreaches, partner facilities and by Community Health Volunteers (CHVs). They would have samples collected and tested on-site (if more than five) or referred to partner facilities for sample collection and testing, or sample referred for testing.

### Data collection and tools

Qualitative data was collected from the relevant stakeholders through key informant interviews using semi-structured interview guides. The interviews were conducted in the respondents’ preferred languages, mainly Swahili, Maasai and English. Quantitative data was extracted from the project records. De-identified patient data was exported from the electronic medical record platform used for the project at the service delivery points and other secondary sources such as the National TB reporting (TIBU) platform. The latter informs the comparative analysis of TB care during the tenure of the project in Kajiado County (May 2021 and January 2023). Variables of interest included Age, Region, Gender, and Mode of Referral. The primary outcome for the study was identification of positive TB cases. Secondary outcomes included identification of extrapulmonary involvement and drug resistant TB.

### Data analysis

All interviews, upon obtaining informed consent, were audio recorded, transcribed, and translated as necessary. Recordings and transcripts were stored on the researcher’s cloud storage systems and accessed only by the investigators.

The quantitative findings were analyzed using descriptive statistics and inferential statistics. Z-Tests for difference of proportions was applied to explore any differences in the project outcomes. Analysis of Variance (ANOVA) was used to compare outcomes during the tenure of the project (May 2021 to January 2023). Multivariate logistic regression with a backward selection approach to identify the best-fitting model was utilized to assess the likelihood of a positive outcome based on gender, age, region and mode of referral.

The qualitative data was organized using Taguette Software. The OECD DAC Evaluation Framework, including Participation and Equity, were adopted for data collection and analysis, guided the deductive thematic analysis of qualitative data. First level analysis of the qualitative data was done through inductive/ open coding. Subsequent level analysis was done through deductive and axial coding with establishment of linkages in keeping with the defined framework. Further, the analysis adopted a flat coding frame with the respective codes having similar significance.

## Results

### Descriptive statistics

#### Cases by age and gender

1350 subjects were presumptive for TB, tested by GeneXpert, and 124 (9.2%) were found positive for TB as shown in Table 2. The evaluation found that while the males were a minority among the presumptive cases (623; 46%), they were significantly (*p*=0.0017) more (59.3%) among the confirmed TB cases compared to females (40.7%) within the ATI TB project in Kajiado County.

**Table 2:** ATI TB project statistics

**Variable**	**Description**	**Presumptive Cases**			**Positive Cases**		
		**Self-Referral**	**Active Case Finding**	**Total**	**Self-Referral**	**Active Case Finding**	**Total**
**Gender**	**Male**	233 (37%)	390 (63%)	623	37(50%)	37(50%)	74
	**Female**	281(39%)	437 (61%)	718	29(58%)	21(42%)	50
	***Sub Total***	***514(38%)***	***827(62%)***	***1341***	***66(53%)***	***58(47%)***	***124***
		^a^Valid Data 1341/1350			^a^Valid Data 124/137		
**Sub County**	**K. West**	428 (57%)	329 (43%)	757	52(66%)	27(34%)	79
	**K. East**	3(2%)	130(98%)	133	3(30%)	7(70%)	10
	**K. North**	47(16%)	256(84%)	303	11(44%)	14(56%)	25
	**K. Central**	0(0%)	22(100%)	22	0(0%)	3 (100%)	3
	**K. South**	1(3%)	29(97%)	30	0(0%)	1(100%)	1
	***Sub Total***	***479(39%)***	***766(61%)***	***1245***	***66(56%)***	***52(44%)***	***118***
		^a^Valid Data 1245/1350			^a^Valid Data 118/137		
**Age**	**0 to 4**	3(50%)	3(50%)	6	0	0	0
	**5 to 9**	18(44%)	23(56%)	41	2(67%)	1(33%)	3
	**10 to 14**	43(38%)	69(62%)	112	3(60%)	2(40%)	5
	**15 to 24**	97(48%)	105(52%)	202	14(67%)	7(33%)	21
	**25 to 34**	116(40%)	178(60%)	294	18(49%)	19(51%)	37
	**35 to 44**	81(39%)	129(61%)	210	12(52%)	11(48%)	23
	**45 to 54**	58(33%)	117(67%)	175	9(53%)	8(47%)	17
	**55 to 64**	42(33%)	86(67%)	128	5(50%)	5(50%)	10
	**65+**	32(34%)	62(66%)	94	3(50%)	3(50%)	6
	***Sub Total***	***490(39%)***	***772(61%)***	***1262***	***66(54%)***	***56(46%)***	***122***
		^a^Valid Data 1262/1350			^a^Valid Data 122/137		

^a^Valid data refers to data appropriately broken down by respective categories i.e., gender, sub-county, and age. ‘Self-Referrals’ as a mode refers to walk in patients at project sites, while Active Case Finding refers to patients who were proactively identified and served within the project courtesy of the outreaches by CHVs, health workers and programme activities; presumptive cases refer to the individuals who have been screened and show symptoms indicative of TB

Approximately 70% of the confirmed TB cases within the project were aged between 15 and 44 years; with those aged between 25- and 34-years accounting for 30% of the cases. The median age of the male patients was 35 years (range between 9 and 74) while among the female patients, it was 29 years (range between 7 and 70). However, there was no significant difference in proportion of confirmed TB from presumptive TB cases among the different age groups (0-4, 5-9, 10-14, 15-24, 25-34, 35-44, 45-54, 55-64, and 65+) (p-value of 0.2854).

#### Regional reach

The respondents noted that the project had improved access to TB diagnosis and care within the county and among the often-marginalized rural communities thus enhancing equity.

Majority of the confirmed cases were from rural Kajiado West (79; 66.9%), followed by Kajiado North (25; 21.2%), Kajiado East (10; 8.5%), Kajiado Central (3; 2.5%), and Kajiado South (1; 0.8%). However, there was not significant difference between the proportion of confirmed TB cases from rural and urban regions (*p*=0.4552).

#### Mode of referral

Though findings of the ATI TB project in Kajiado indicate that 61% of the presumptive cases were through ACF, only 7% of these tested positive. Conversely, 13% of the self-referrals (39% of the presumptive) tested positive.

Cumulatively, approximately 66 (53%) of the positive cases with valid data were self-referrals while ACF through CHV referrals and outreaches accounted for (58) 47% of the positives. 13 of the positive cases had incomplete data hence excluded in this summation. The differences in proportion of confirmed TB cases from Self Referrals and ACF were significant (*p*=0.0003).

Multivariate logistic regression analysis was used to ascertain the association between positive diagnostic outcome and; age, gender, region, and mode of referral. Only gender (β=8.76, *P*=0.0003) and mode of referral (β=23.86, *p*<0.0001) were significant predictors of test outcome using backward selection. The odds of being positive were 186 % higher for self-referral as compared to ACF; and 91% higher among males than females.

#### ACF of Extrapulmonary and Drug Resistant (DR) TB

Majority of the confirmed TB cases (94%) had pulmonary involvement while 6% had extrapulmonary involvement. There was no significant difference between the proportion of extrapulmonary TB cases in the project and those (9%) notified in the county (*p*=0.437).

Comparatively, the DR TB cases 4 (3%) were significantly higher within the project than in the county 28 (1%) as captured in the TIBU platform [30] (*p*=0.025).

#### Patient-centred care

Most respondents noted that ATI TB adopted an integrated approach in the management of TB, was inclusive and respected the beneficiaries dignity, beliefs and cultures. A rural community representative remarked that *“The project respected the culture. There was learning, most of the time they would come and enter into the community and homes and request to do the screening and their culture notwithstanding, the residents would agree…”*

Respondents highlighted poverty and financial disparity as barriers to accessing care within Kajiado and noted that offering free TB services improved the residents' health-seeking behavior. The food incentives given to those that tested positive also motivated them to comply with the treatment. For instance, one rural patient representative said *“The project has also saved the community huge amounts of finances spent on buying drugs by giving us drugs for free.”*

Most of the respondents commended the integration of TB care with other disease control programs as this improved efficiency, reduced stigma and maximized value for the communities. One rural patient representative noted* “…reaching out to patients in their villages and offering them drugs not only for TB but also for other diseases and in this way, patients and the local communities benefit the most.”*

#### Environmental awareness

The respondents noted that the project improved environmental awareness since they were educated on the importance of maintaining a clean environment to curb the spread of TB and other ailments and taught on the importance of reforestation. As such, one Community Health Volunteer remarked “*Environmentally, they have taught the residents that its airborne and so they should improve on the aeration, in relation to this even the manyattas (traditional houses) have been well ventilated after the awareness.”*

#### Political engagement

The respondents highlighted the value of government collaboration to ensure adequate and motivated health-workers and increased funding for continued ACF and timely management of TB. For instance, one Government representative noted that *“The Impact will last. The county government will now step in and ...will be conducting the same activities in the same areas”.*

#### Project impediments

The respondents noted the need for increased government investments in health systems strengthening within Kajiado County. The following were noted impediments to the project implementation: a) Stigma, b) limited awareness among stakeholders on quality TB care, c) disruption of care due to limited availability of critical inputs e.g., GeneXpert Machines, test cartridges, d) intermittency of the services provided through outreaches, e) weak county governance and bureaucracies, g) inadequate staffing, and h) weak stakeholder collaboration.

#### Evaluation

**Fig. 2:**
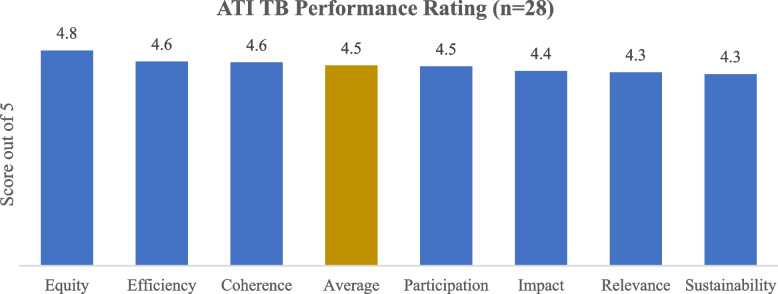
Subjective assessment of the project

Subjective assessment of the thematic dimensions of the project under the OECD DAC framework demonstrated a high level of satisfaction by the sampled stakeholders with an aggregate average of 4.5 out of 5 (*n*=28) as depicted in Fig. 2. Notably, most stakeholders sampled ranked Equity highest (4.8 out of 5), followed by Coherence and Efficiency (4.6 out 5), Participation (4.5 out of 5), Impact (4.4 out of 5) and Sustainability at 4.3 out of 5.

##### Relevance

The respondents highlighted the project's value in improving access to quality, responsive and patient-centred TB care in Kajiado County as was captured in the comment by one urban community representative *“At the end of the day they also do the home visits, when they do the home visit in a company of CHV, they do the screening and also after the screening they also refer the clients to the facility so that they can be able to initiate treatment”*.

##### Coherence

The respondents felt that the project ensured alignment to global and national best practice and priorities with respect to championing Active Case Finding of TB, increasing Bacteriological confirmation over Clinical diagnosis of TB, and enhancing strategic collaboration through Public Private Mix (PPM) in TB care. A county government official stated that *“...they are using the same guidelines from the National TB program” and further added that “..this project is also in line with national strategic plan whereby we aim at getting cases; diagnosing of TB, getting more patients on treatment as much as possible, reaching to the community to ensure we get the missed TB in the community.”*

##### Equity

Most of the beneficiaries were populations found in the remote and rural areas of Kajiado, and those in informal settlements within Kajiado; all of whom are vulnerable populations by virtue of their socio-economic circumstances. A patient from the urban communities noted that *“The poor, the differently abled, women and the ethnic minority are all treated and given the same services without any form of discrimination.” Further, one project staff said “... we could go very far to do screening and testing on site which was beneficial to the communities. It relieved them of coming from rural to the urban to get healthcare and that eased expenditure on transport.”*

##### Effectiveness

The respondents also pointed out that the project had improved treatment adherence among the patients found to be positive in both urban and rural regions of Kajiado County as all who were found to be positive were put on treatment. One rural patient beneficiary stated *“I am so happy because through this project I have never gone short of drugs which has always been my top priority”. A project staff noted that “It (the project) has responded to their (Kajiado Community) needs because if you see the number of positives we have gotten, more than a hundred. It is a big number and three-quarters of that comes from the rural areas so it’s really a boost for them.”*

##### Efficiency

The respondents noted that the project enhanced efficient use of existing resources (human, technological and financial) within the project sites in Kajiado County. This was through integration of TB services with other critical primary care services such as Reproductive, Maternal, Newborn and Child Health services, targeted screening among the populations deemed vulnerable in Kajiado County, improved diagnostic capacity for existing care providers, improved linkage of care through patient and sample referrals as deemed appropriate, resource redistribution through TB outreaches inter alia. A few such affirmations included *“GeneXpert machine has also made sure we receive results of the screening as fast as possible”* from an Urban Patient Beneficiary and *“...they no longer need to travel to Nairobi or distant places to get the machines for TB testing, everything is now done in Oltepesi in a very simple and cost-effective way” by one Rural Patient Beneficiary.*

##### Impact

While the project primarily sought to improve Active Case Finding for TB, and improve stakeholder awareness on TB care, respondents noted that it resulted in other non-TB care related effects including improved nutrition of patients, improved financial protection for the vulnerable poor, improved access to other health services such as Antenatal Care and primary care services for the remote population, economic empowerment in the communities and environmental management awareness. One patient said *“I have been on drugs for six months and have also received a food package for the entire time as a patient on dugs”. Another rural patient mentioned that “…reaching out to patients in their villages and offering them drugs not only for TB but also for other diseases and in this way, patients and the local communities benefit the most.”* Further, another rural patient noted that *“We have also received food packages and this has enabled our children to avoid hunger and be able to go to school.”*

##### Sustainability

Through collaboration and strategic partnerships with National programs, county government and private hospitals, the respondents strongly felt that the effects of the project would continue and outlast the project. A representative of the rural community noted that *“There is this support you gave in the form of GeneXpert machine and even after you are gone, the machine will still be serving the whole community In Kajiado West and that is sustainability.”*

##### Participation

Majority of the respondents noted that the project had significantly allowed for participation of the stakeholders in the design, and implementation of the interventions which engendered in goodwill and ownership. Notably, the following were involved: county and community leadership, traditional healers, patients, health workers, public and private sector collaborators. A private sector partner said that *“It was teamwork. We worked together as a whole team, the partners, the donors, everything went smoothly. Everyone was involved, actively involved.”* A community member also noted that *“…they (ATI TB team) also engaged the traditional healers, bearing in mind Kajiado, we have people who seek services from the traditional healers and herbalists.”*

## Discussion

This study demonstrates the outcomes of the Awareness, Traditions, and Innovations (ATI) in TB project on improving access to quality TB care in Kajiado County. We found that the burden of all forms of TB was higher among males in Kajiado (59.3%; *p*=0.0017) with more positive TB cases among the self-referrals (13%) compared to those identified via ACF (7%; *p*=0.0003). The findings also suggest that the project increased the potential for identification of Drug Resistant TB (3% vs 1%; *p*=0.025) more than passive approaches at county level. The study respondents posit that integrated and Patient centered care approaches that not only target TB but address other determinants of health are more likely to yield better engagement, ownership, impact, and sustainability.

The study showed that the burden of all forms of TB was higher among males in Kajiado County (59.3%; *p*=0.0017). This aligns with county level evidence of rising cases of pulmonary and extrapulmonary TB in males from 65% to 69%, and 54% to 55% respectively as recorded in the TIBU platform [30]. The Kenya National TB Prevalence Survey [9] and a subsequent nationwide cross-sectional survey by Masini et al 2018 [10] also found a higher burden of TB among the males than females. Sathiyamoorthy et al, 2020, also found that the prevalence in India varied based on sex and was higher among males than females [31]. The higher burden among males may be influenced by the poor health seeking practices and attitudes that esteem care seeking among males as a sign of weakness; socio-economic obligations on the males *inter alia*. This study reinforces the findings on higher disease burden among males and adds the contextual subnational level finding on the burden of TB among the males in Kajiado County.

A higher proportion of the positive cases within the project (66.7%) were from the rural areas in Kajiado (Kajiado West). This finding differs from the National TB survey which suggests a higher burden of TB in the urban areas compared to the rural areas [9, 10]. However, this finding agrees with evidence from Sathiyamoorthy et al, 2020 in India who found that the prevalence of TB varied also based on regions, with a higher burden in the rural regions [31]. The findings in the project may have been skewed by the project’s focus on ACF in the rural areas. These findings highlight the potential for increasing access to TB care through ACF in rural areas within decentralized health systems.

The study shows more positive TB cases among the self-referrals compared to those identified via ACF (13% vs 6%; *p*=0.0003). The findings also suggest that ACF can increase the potential for identification of Drug Resistant TB more than passive approaches (3% vs 1%; *p*=0.025). These findings underscore the reality of unmet care needs at community level, most of which are underdiagnosed and under reported given that less than 31% of facilities have TB diagnostic capacity in Kenya [16]. These agree with the existing evidence on higher burden of disease than is diagnosed in most systems and the significance of ACF in strengthening care [9, 10, 14, 25, 26, 31].

The study finds that 6% of the cases had extrapulmonary involvement. This is slightly lower than the county level burden of 9% as notified on TIBU [30]. Subbaraman et al, 2016, point out the tendency to poor outcomes among the patients with Drug Resistant TB [32], as do Banta et al, 2020 on Extrapulmonary TB and recommend improved methods for screening, diagnosis and treatment [33].

The study respondents posit that integrated and patient-centered approaches that not only target TB but address other determinants of health (socio-cultural, economic, environmental, political) are more likely to yield better engagement, ownership, impact and sustainability. This is consistent with evidence on the role of patient centred approaches that recognize and respect individual and community health needs, values, preferences and expectations rather than disease [22–25, 27, 28]. Ruiz-Tornero et al 2022, emphasize that social and economic factors not only influence the incidence of TB, diagnosis, and treatment but also determine the approach to care [34]. This study provides contextual insights on the value of patient centred, decentralized, and integrated approaches for sustainability in TB care. It also highlights local systemic drivers of health inequity in Kajiado.

### Generalizability and Limitations

The project’s reach was limited to 4 of the 5 sub-counties in Kajiado; was heavily targeted at the rural sub-counties, and to a limited sample, purposively recruited. Consequently, these limit the generalizability of the findings due to sample size and frame. Additionally, assessing the impact of ACF for TB across a broader population including urban areas in Kajiado County remains a potential opportunity for future research.

The evaluation could not draw direct attribution of some outcomes such as changes to the prevalence, incidence, mortality of TB across the entire Kajiado County to the project given the multiple external health system and non-health factors that influence these indicators.

### Recommendations

The following are the recommendations based on key the findings:

1. Increased and targeted TB sensitization and screening among the vulnerable groups including males and rural residents.
2. Patient education on the importance of adherence to treatment to minimize drug resistance and extra pulmonary TB relapses.
3. Advocacy for patient centered care, keeping in mind the sick individual while designing strategies geared toward TB care.
4. Capacity development among health workers for improved diagnosis, care, and treatment for extra-pulmonary TB and Drug Resistant TB.
5. Scaling up of ACF for TB across the county.
6. Continued training and support to the CHVs in ACF.
7. Increased Government Investment in TB care and health system strengthening.

## Conclusions

Stemming the rising burden of TB necessitates sustained, proactive, and concerted action by all stakeholders with a view to increasing awareness; improving screening and diagnosis through ACF; adoption of integrated and comprehensive patient centred care approaches; and increased investments in the ecosystem that supports health at community level.

### Supplementary Information

**Additional file 1.** 

**Additional file 2.** 

**Additional file 3.** 

## Data Availability

The authors confirm that the data supporting the findings of this study are available within the article and its supplementary materials.
